# An Exploratory Study of Machine Learning-Based Open-Angle Glaucoma Detection Using Specific Autoantibodies

**DOI:** 10.3390/biomedicines13123031

**Published:** 2025-12-10

**Authors:** Naoko Takada, Makoto Ishikawa, Takahiro Ninomiya, Yukitoshi Izumi, Kota Sato, Hiroshi Kunikata, Yu Yokoyama, Satoru Tsuda, Eriko Fukuda, Kei Yamaguchi, Chihiro Ono, Tomoko Kirihara, Chie Shintani, Akiko Hanyuda, Naoki Goshima, Charles F. Zorumski, Toru Nakazawa

**Affiliations:** 1 Department of Ophthalmology, Tohoku University Graduate School of Medicine, Sendai 980-8574, Japan; naoko.takada.d7@tohoku.ac.jp (N.T.); makoto.ishikawa.c2@tohoku.ac.jp (M.I.);; 2 Seiryo Eye Clinic, Sendai 980-0824, Japan; 3 Taylor Family Institute for Innovative Psychiatric Research, Washington University School of Medicine, St. Louis, MO 63110, USA; 4 Center for Brain Research in Mood Disorders, Washington University School of Medicine, St. Louis, MO 63110, USA; 5 Department of Psychiatry, Washington University School of Medicine, St. Louis, MO 63110, USA; 6 Department of Advanced Ophthalmic Medicine, Tohoku University Graduate School of Medicine, Sendai 980-8574, Japan; 7 Department of Retinal Disease Control, Ophthalmology, Tohoku University Graduate School of Medicine, Sendai 980-8574, Japan; 8 ProteoBridge Co., Tokyo 135-0064, Japan; 9 Molecular Profiling Research Center for Drug Discovery, National Institute of Advanced Industrial Science and Technology (AIST), Tokyo 135-0064, Japan; 10 Cellular and Molecular Biotechnology Research Institute, National Institute of Advanced Industrial Science and Technology (AIST), Tsukuba 305-8566, Japan; 11 Ophthalmic Innovation Center, Santen Pharmaceutical Co., Ltd., Osaka 530-0011, Japan; 12 Product Development Division, Santen Pharmaceutical Co., Ltd., Nara 630-0101, Japan; 13 Department of Ophthalmology, School of Medicine, Keio University, Shinjuku-ku 160-8582, Japan; 14 Epidemiology and Prevention Group, Center for Public Health Sciences, National Cancer Center, Tokyo 104-0045, Japan

**Keywords:** autoantibody, open-angle glaucoma, diagnosis, machine learning

## Abstract

**Objectives:** Previously, we identified four open-angle glaucoma (OAG)-associated autoantibodies (anti-ETNK1, anti-VMAC, anti-NEXN, and anti-SUN1) using proteome-wide autoantibody screening by wet protein arrays. The objective of this exploratory study was to evaluate the diagnostic performance of these four glaucoma-associated autoantibodies using automated machine learning. **Methods:** Plasma samples from 119 patients with OAG and 35 patients with cataracts as controls were enrolled for the study. All machine-learning analyses were performed in Python 3.9.16 (GCC 11.2.0) using scikit-learn 1.2.2 and PyCaret 3.0.1. Variables included plasma levels of the autoantibodies, age, sex, and intra-ocular pressure (IOP). Probability calibration (Platt/sigmoid and isotonic) was assessed with reliability curves and Brier scores. Model explainability was examined with permutation importance, SHAP values, and an ablation analysis removing one autoantibody at a time. **Results:** The tuned random forest achieved an out-of-fold (OOF) area under the receiver-operating characteristic curve (ROC–AUC) of 0.852 (±0.040), an average precision (AP) of 0.950, and an F1 score of 0.865. Isotonic mapping improved agreement between predicted and empirical probabilities. Among these four autoantibodies, VMAC was the most important factor for the model’s prediction. **Conclusions:** A machine learning model using four autoantibodies from blood samples showed potential for diagnosing OAG.

## 1. Introduction

Glaucoma is one of the leading causes of blindness in adults [1]. Many cases of glaucoma progress gradually without obvious symptoms until the late stages of illness [2]. By the time statistically significant abnormalities become detectable on automated perimetry, at least 25–35% of retinal ganglion cells have already been lost [3]. Early detection and treatment are essential [4]. However, there is no simple objective screening test for glaucoma. Only an ophthalmologist or optometrist can determine whether an individual has glaucoma by performing an intra-ocular pressure (IOP) measurement, testing visual fields, taking images or computer measurements of the optic nerve. It is, thus, important to develop objective and simple screening methods that do not rely on such complex clinical eye examinations.

Autoantibodies have been reported in autoimmune diseases [5], infectious diseases [6], and malignant tumors [7]. Recently, there has been increasing evidence supporting the involvement of autoimmune reactions in glaucoma [8,9,10,11,12,13,14,15,16,17,18,19,20,21]. In our previous study [22], we performed a comprehensive analysis of plasma autoantibodies in patients with glaucoma using wet protein arrays (WPA) [23,24,25,26] and found that antibodies against ethanolamine kinase 1 (ETNK1), vimentin-type intermediate filament-associated coiled-coil protein (VMAC), nexilin (NEXN), and Sad1 and UNC84 Domain-Containing 1 (SUN1) are glaucoma-associated autoantibodies.

Machine learning, a subfield of artificial intelligence, encompasses classical algorithms and more advanced deep learning methods. In classical machine learning, the model is initially trained using the correct answers provided. Classical machine learning algorithms are designed based on mathematical algorithms such as linear regression, logistic regression, decision trees, Support Vector Machine (SVM), and others. Examples of classical machine learning include cancer diagnosis through analysis of MRI or CT images [27,28]. By training the model using pre-existing diagnostic data from physicians as labeled data, AI models can be built to rapidly identify disease risks from images. Classical machine learning achieves high prediction accuracy even with relatively small datasets. On the other hand, deep learning automatically extracts features using multi-layer neural networks, which is a machine learning model that processes data by mimicking the way the human brain works. By learning important features directly from the input data and acquiring hierarchical representations, deep learning models can capture complex patterns that may not be noticed by humans. This capability enables these models to achieve high accuracy in domains that are difficult for humans to process or explicitly define, including images, audio, and natural language analysis. In recent years, numerous studies have reported the high diagnostic performance of deep learning approaches for glaucoma detection using fundus photographs [29,30], optical coherence tomography (OCT) [31,32] or visual field tests [33]. Notably, while deep learning excels at analyzing big data, it is less suitable for analyzing relatively small datasets. For AI-based glaucoma prediction using autoantibodies, classical machine learning is considered more appropriate than deep learning [34].

To date, there have been only a few studies using machine learning techniques to analyze biomarkers, including autoantibodies in patients’ blood to predict glaucoma [19,21,35,36]. In the present exploratory study, we hypothesized that machine learning algorithms could identify specific patterns of autoantibodies in the plasma of open-angle glaucoma (OAG) patients. We applied automated machine learning using the plasma levels of these autoantibodies in our previously reported dataset [22] to evaluate their diagnostic performance for OAG. These approaches may facilitate predicting the most likely diagnosis for OAG patients.

## 2. Methods

### 2.1. Subjects

This study used our previously reported dataset [22]. The research method is a retrospective case–control design and was approved by the Ethics Committee of Tohoku University (2022-1-831). Patients who attended the Department of Ophthalmology of Tohoku University Hospital from March 2015 to May 2018 were included in this study after providing full written informed consent in accordance with the Declaration of Helsinki on Medical Research Involving Human Subjects. Blood tests and ophthalmologic examinations were performed. Patient information, symptoms, medications, and laboratory findings at the closest point in time to the date of blood tests were collected retrospectively from electronic medical record information. The OAG group included patients with mild or early (mean deviation (MD) ≥ −6 dB; 11.8%) and progressive (MD < −6 dB; 88.2%) glaucomatous visual field loss. Among the OAG cases, 50 were classified as normal-tension glaucoma (NTG), while 69 were classified as high-tension glaucoma (HTG). The control group consisted of age- and sex-matched cataract patients without other ocular diseases, including glaucoma. IOP, fundus photographs, and OCT imaging tests were referenced to exclude ocular diseases other than glaucoma and cataract. OAG cases and controls were excluded if they self-reported a history of autoimmune diseases, cancers, or internal ocular surgery within the past year, as these conditions could affect blood autoantibodies. Other systemic diseases were not excluded; subjects with glaucoma types other than NTG and HTG were excluded. Statistical methods were not used to predetermine the sample size as this was an exploratory study.

### 2.2. Wet Proteome Analysis

A proteome-wide autoantibody screening method was used as previously outlined [24]. First, 19,446 human proteins for antigens were synthesized from HuPEX (Human Proteome Expression Resource) entry clones of the entire human proteome cDNA library [22]. pEW-5FG with SP6 promoter and N-terminal FLAG-GST tag was used as the destination vector, and protein synthesis was performed using the wheat germ cell-free translation system (CellFree Sciences Co., Ltd., Matsuyama, Japan) [22,23]. Second, human proteins were spotted onto the plate using a HORNET-NX multi-dispense system equipped with a 1536-pin head (Fujifilm Wako Pure Chemical Co., Osaka, Japan) and bound via their GST-tags [24].

The next step was quantification of autoantibodies in patient plasma of each disease type by a custom-designed WPA. A glutathione (GSH)-modified glass plate (SDM0011, Matsunami Glass Industry Co., Ltd., Kishiwada, Japan) was used for a custom-designed wet protein array (WPA). GST-tag fused human proteins were spotted using a 1536-channel independent cylinder system (EDR-384SX; Biotec, Bunkyoku, Japan). The target antigens of the autoantibody were spotted on a custom-designed WPA.

For quantification of autoantibodies, the level of each autoantibody was calculated based on the fluorescence values obtained from the reaction of plasma with the protein spot [12]. The level of each autoantibody was calculated as autoantibody titer (in arbitrary units, AU) and positivity rate (PR).

### 2.3. Machine-Learning Methods

All machine-learning analyses were performed in Python 3.9.16 (GCC 11.2.0) using scikit-learn 1.2.2 and, for exploratory screening only, PyCaret 3.0.1. After harmonizing labels and applying uniform quality control, essential predictors included four autoantibodies (NEXN, SUN1, ETNK1, VMAC), age, sex, and IOP; sex was encoded as 0 for female and 1 for male. The binary outcome variable was encoded as 0 (cataract) and 1 (OAG). Machine learning algorithms can generally be categorized as supervised or unsupervised, depending on whether labeled outcome data are used for training. Because the present study aimed to classify subjects with and without OAG based on labeled clinical and serological data, a supervised learning approach was employed. A transparent scikit-learn pipeline was used throughout: median imputation and z-score standardization were applied within a Column Transformer, and evaluation relied on stratified 5-fold out-of-fold (OOF) predictions to avoid optimistic bias.

#### 2.3.1. Exploratory Model Comparison

Exploratory model comparison was initially performed using PyCaret; however, anomalous AUC outputs were observed in several models. Therefore, all sixteen classical classifiers were re-implemented and benchmarked in scikit-learn to ensure full transparency and reproducibility under identical preprocessing and OOF evaluation. The evaluated classifiers included: logistic regression, ridge classifier, linear discriminant analysis, k-nearest neighbors, Gaussian naïve Bayes, support-vector machine (linear and radial-basis-function kernels), decision tree, random forest, extra-trees, gradient boosting, extreme gradient boosting (XGBoost), CatBoost, light gradient boosting machine (LightGBM), AdaBoost, and quadratic discriminant analysis. A dummy classifier (uniform random prediction) was included as an uninformed baseline. All models were evaluated using “predict_proba” or “decision_function” as appropriate and ranked by mean OOF area under the receiver-operating characteristic curve (ROC–AUC). The random-forest algorithm was selected for subsequent optimization and analysis due to its consistently high internal performance in preliminary screening.

#### 2.3.2. Random-Forest Optimization

A Random Forest Classifier (sklearn.ensemble) was tuned by grid search over n_estimators ∈ {50, 100, 200, 400} and max_features ∈ {“sqrt”, 2, 4, None}, max_depth ∈ {None, 3, 5, 7}, and min_samples_leaf ∈ {1, 2, 4}, maximizing OOF ROC–AUC with stratified 5-fold cross-validation and a fixed random state for reproducibility. All preprocessing was implemented within the same transparent scikit-learn pipeline: missing values were imputed by the median, and continuous variables were standardized using z-score normalization via a ColumnTransformer. The tuned model was then used to generate fold-wise OOF predictions from which discrimination metrics were computed, including ROC-AUC and average precision (AP). To ensure probability reliability, we assessed calibration using Platt (sigmoid) and isotonic mappings via CalibratedClassifierCV and summarized reliability curves together with Brier scores. Model explainability combined permutation importance (10 repeats), and SHapley Additive exPlanations (SHAP) values (TreeExplainer); an ablation analysis removed one autoantibody at a time while keeping the remaining predictors and the same CV protocol.

The best-performing random-forest configuration obtained from grid search used n_estimators = 50, max_features = 4, max_depth = 3, and min_samples_leaf = 4, and this tuned model was used for all subsequent analyses.

### 2.4. Statistical Analysis

All analyses were performed using Python 3.9.16 (scikit-learn) and R version 4.1.2. In addition, *t*-tests and Fisher’s exact test were used to compare the backgrounds of the subjects. Primary discrimination metrics (ROC–AUC and AP) were derived from stratified 5-fold OOF predictions. Ninety-five percent confidence bands for ROC curves were obtained by nonparametric bootstrapping of the OOF prediction–label pairs. Where applicable, DeLong tests were performed in R for AUC comparisons. Two-tailed *p* values < 0.05 were considered statistically significant for baseline comparisons.

## 3. Results

Plasma samples were obtained from 35 patients with cataract, 119 patients with OAG, and a custom-designed WPA analysis containing a total of 220 antigens was conducted. ETNK1, VMAC, NEXN, and SUN1 were identified as glaucoma-associated autoantibodies based on their plasma levels and positive rates [22].

Machine learning analysis was conducted to classify OAG versus control subjects using the following variables: the four plasma levels of autoantibodies (ETNK1, VMAC, NEXN, and SUN1), age, sex, and IOP (Table 1).

In an internal scikit-learn benchmarking across sixteen classical classifiers under identical preprocessing, random forest ranked among the top performers (see Table 2 for the top five models; see full results in Supplementary Table S1).

**Figure 1:**
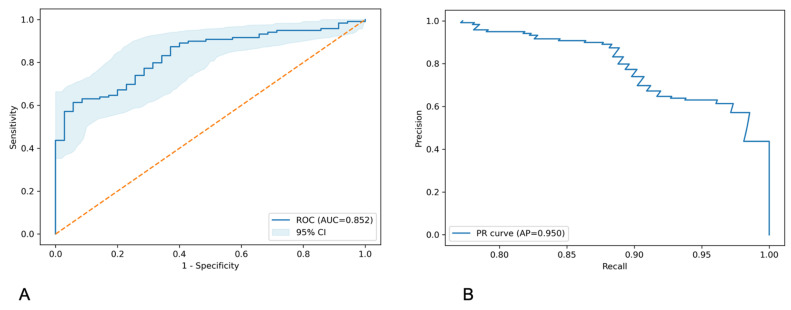
Discrimination performance of the tuned random-forest model using stratified five-fold OOF evaluation. (**A**): ROC curve with a bootstrap-based 95% confidence band drawn from pooled OOF predictions (AUC = 0.852 ± 0.04) (95%CI: 0.76–0.90). The corresponding fold-wise mean AUC was 0.839 ± 0.026 (Table 2). Orange dashed diagonal lines indicate the reference line for a non-discriminative classifier (AUC = 0.5), where sensitivity equals 1 − specificity. (**B**): PR curve from the same OOF predictions, showing high AP of 0.950 and favorable precision–recall trade-offs. OOF = Out-Of-Fold; ROC = Receiver-Operating Characteristic curve; AUC = Area Under the Curve; PR = Precision–recall; AP = Average Precision.

Based on this preliminary benchmarking, the random-forest model was selected for subsequent optimization and evaluation. Using a transparent scikit-learn pipeline with median imputation, z-score standardization, and stratified five-fold OOF evaluation, the model achieved an OOF AUC of 0.839 ± 0.026 (5-fold mean) and an average precision of 0.950. After hyperparameter optimization, the tuned random forest with all seven predictors achieved a higher mean OOF ROC–AUC of 0.852 ± 0.040 (95%CI:0.76–0.90) (Supplementary Tables S2 and S3). The ROC curve shown in Figure 1A was derived from pooled OOF predictions and yielded a numerically similar AUC of 0.852, which was expected to differ slightly from the fold-wise mean due to averaging versus pooling. In addition, these results indicated strong discrimination and favorable precision–recall trade-offs (Figure 1A,B). The ROC curve was displayed with a bootstrap-based 95% confidence band.

**Figure 2:**
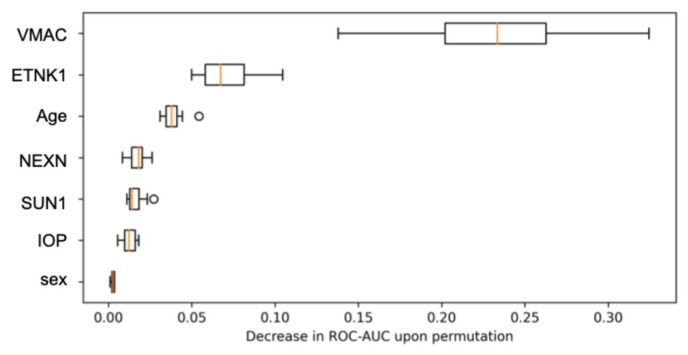
Feature contributions based on permutation importance. The contribution of each autoantibody, age, IOP, and sex to the model prediction by Permutation Importance. VMAC and ETNK1 showed the largest decreases in ROC–AUC upon permutation, indicating their dominant contributions to model performance. VMAC = vimentin-type intermediate filament-associated coiled-coil protein; ETNK1 = ethanolamine kinase 1; NEXN = nexilin; SUN1 = Sad1 and UNC84 domain-containing 1; IOP = intra-ocular pressure; ROC–AUC = area under the receiver-operating characteristic curve.

Permutation-based feature importance showed the largest mean AUC decrease when permuting VMAC, followed by ETNK1 and age, whereas NEXN and SUN1 had modest effects, and IOP and sex had minimal effects (Figure 2).

An ablation analysis (removing one autoantibody at a time under the same OOF protocol) confirmed the largest AUC drop for VMAC removal, followed by ETNK1 (Supplementary Table S2).

**Figure 3:**
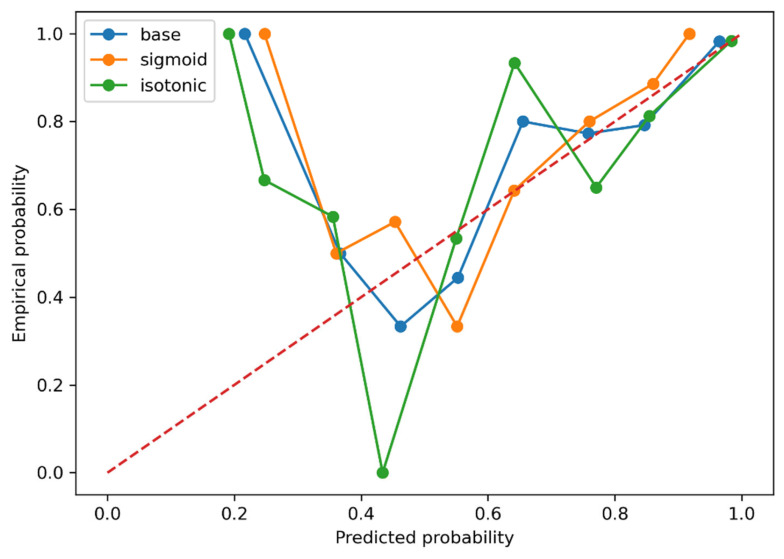
Calibration of predicted probabilities. Calibration plots for the base (uncalibrated), sigmoid, and isotonic models. The red dashed line indicates perfect calibration. Both sigmoid and isotonic calibration produced similar overall Brier scores and discrimination to the uncalibrated model, while isotonic mapping improved the visual alignment in the high-probability range.

Reliability analysis demonstrated reasonable agreement between predicted and empirical probabilities; isotonic calibration improved alignment in the high-probability range with minimal change in discrimination (Figure 3 and Supplementary Table S3).

**Figure 4:**
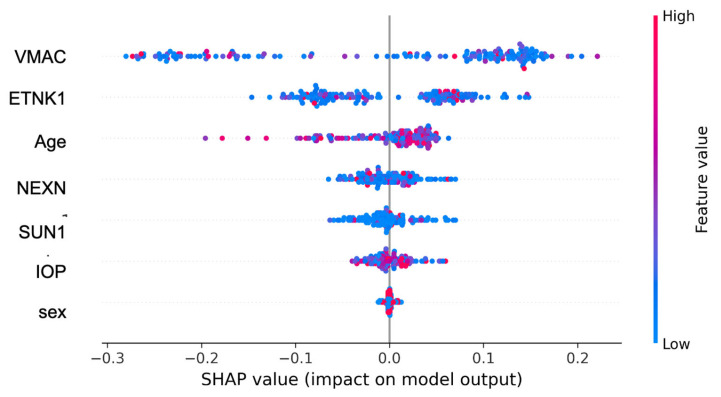
SHAP beeswarm plot showing sample-wise contributions. Each dot represents a sample, with color indicating the feature value (red = high, blue = low). Higher VMAC and ETNK1 values were associated with increased OAG prediction probability. VMAC = vimentin-type intermediate filament-associated coiled-coil protein; ETNK1 = ethanolamine kinase 1; NEXN = nexilin; SUN1 = Sad1 and UNC84 domain-containing 1; IOP = intra-ocular pressure; OAG = open-angle glaucoma.

SHAP beeswarm plots illustrated sample-wise directionality consistent with permutation results, with higher VMAC and ETNK1 values pushing predictions toward OAG (Figure 4). A complementary bar-type SHAP summary of global feature importance was provided in Supplementary Figure S1.

## 4. Discussion

In this exploratory study, using four plasma autoantibodies against ETNK1, VMAC, NEXN, and SUN1 [8,20], we developed an AI model for automated OAG diagnosis using random forest machine learning. The training model was evaluated using scikit-learn under a stratified five-fold OOF protocol and yielded an OOF AUC of 0.852 ± 0.040 and an AP of 0.950, indicating robust discrimination and favorable precision–recall performance. VMAC was identified as the most influential factor in model prediction. Permutation importance and SHAP analyses consistently highlighted VMAC and ETNK1, and an ablation analysis confirmed the largest AUC drop when removing VMAC (ΔAUC = −0.10).

The model was developed by initially using sixteen candidate algorithms (logistic regression, ridge classifier, linear discriminant analysis, k-nearest neighbors, naïve Bayes, linear-kernel support-vector machine, decision tree, random forest, extra-trees, gradient boosting, light gradient boosting machine (LightGBM), extreme gradient boosting (XGBoost), AdaBoost, quadratic discriminant analysis, and CatBoost) to predict OAG diagnosis using four plasma autoantibodies. After comparing all models, random forest demonstrated the best overall discrimination by OOF ROC–AUC, with CatBoost and Extra Trees following (Table 2). Considering that the purpose of this study is glaucoma screening, it can be concluded that the random forest model, which shows high accuracy and recall, is the most suitable. In addition, based on the F1 score and MCC, CatBoost and Extra Trees can also be regarded as well-balanced models. This result is consistent with findings on aqueous humor immune mediators reported in previous research [33]. This suggests that the characteristics of random forest may align well with the nature of autoantibodies. Random forest uses multiple decision trees trained on random subsets of features to capture important characteristics while avoiding overfitting. Other machine learning models exhibited several important drawbacks including the possibility that specific features may be emphasized (ridge classifier), computational complexity increases significantly with larger datasets (k-nearest neighbors), inability to capture relationships between nonlinear features (linear-kernel support-vector machine), susceptibility to overfitting (decision tree), or performance degradation when features are correlated (naïve Bayes), among others. Thus, the Random Forest model was identified as the most suitable approach for the objectives of this study. Probability calibration using isotonic mapping improved agreement between predicted and empirical probabilities in the high-probability range, while overall discrimination and Brier scores remained similar to the uncalibrated model. The results of this study provide evidence that applying machine learning algorithms to expression of plasma autoantibodies can predict OAG.

In recent years, numerous studies have attempted to use AI to diagnose glaucoma based on imaging data [29,30,31] or biomarkers [19,21,37]. The AI model for OAG diagnosis based on autoantibodies achieved diagnostic accuracy comparable to that of previous biomarker-based models [19,21]. However, since imaging-based diagnostic models demonstrate higher accuracy than biomarker-based models, there is a need to further improve the diagnostic performance of biomarker-based approaches. While glaucoma detection models previously focused on single modalities [38], the field is increasingly incorporating multimodal data in algorithms to improve model performance and better align with clinical practice [39]. Multimodal analysis incorporating imaging data and biomarkers is expected to enhance the accuracy of AI-based glaucoma diagnosis [39]. Even though the accuracy of glaucoma diagnosis through single-modal AI analysis of biomarkers alone requires improvement going forward, it is important to develop a predictive model that integrates AI analysis with biomarkers, including autoantibodies and retinal imaging, to enhance illness detection.

It is also important to elucidate the relationship between the identified biomarkers and the pathophysiology of glaucoma. As shown in our previous work, the four autoantibodies did not exhibit significant correlations with ophthalmic parameters (e.g., IOP, central corneal thickness, visual field mean deviation, axial length), although prior studies have suggested potential pathophysiological relevance [22]. In particular, VMAC was the autoantibody that contributed most to our glaucoma diagnostic model. VMAC is indirectly associated with vimentin-type intermediate filaments via vimentin-binding proteins. The presence of autoantibodies to vimentin has been previously reported in patients with NTG. Since elevated IOP increases vimentin synthesis in astrocytes [40], VMAC may be associated with glaucomatous damage caused by elevated IOP.

This exploratory study has several limitations. The sample size was relatively small (*n* = 154; 119 OAG and 35 controls), and the retrospective, single-center design may introduce selection bias. In addition, because the autoantibody testing platforms used in this study are not commercially available, external validation using independent datasets could not be conducted at this stage. These constraints may limit the generalizability of our findings. To address these issues, we plan to evaluate the external validity of the proposed diagnostic model in larger prospective multicenter cohort studies once commercially standardized assays become accessible and ethically shareable datasets can be generated. Furthermore, we aim to develop a multimodal diagnostic approach by integrating widely obtainable health-check data, such as fundus photographs and routine biochemical laboratory parameters, to enhance predictive performance and facilitate real-world clinical applicability. We believe that these future directions will contribute to the clinical implementation and further advancement of autoantibody-based biomarkers for glaucoma diagnosis.

## 5. Conclusions

A machine learning model for OAG diagnosis was developed using a panel of plasma autoantibodies. The results suggest that autoantibody-based biomarkers may serve as useful adjuncts for glaucoma screening. Further validation in larger, multi-center cohorts is required to enhance clinical applicability.

## 6. Patents

Tohoku University, ProteoBridge, Santen Pharmaceutical Co., Ltd., Diagnosis of glaucoma, P2023-129215A, Japan Patent Office, JPO, 2023.

## Figures and Tables

**Table 1:** Characteristics of each study population. The clinical characteristics of the patients from whom specimens were obtained were statistically compared with each other. Cat = cataract; OAG = open-angle glaucoma; IOP = intra-ocular pressure; ETNK1 = ethanolamine kinase 1; VMAC = vimentin-type intermediate filament-associated coiled-coil protein; NEXN = nexilin; and SUN1 = Sad1 and UNC84 domain-containing 1.

	Cat	OAG	*p* Value
N	35	119	
Age (mean ± SD)	69.97 ± 10.84	68.76 ± 7.03	0.433
Sex = Male (%)	18 ± 51.4	68 ± 57.1	0.686
IOP (mean ± SD)	14.06 ± 3.31	14.54 ± 4.43	0.553
ETNK1 (mean ± SD)	0.83 ± 1.05	3.93 ± 16.79	0.047
VMAC (mean ± SD)	0.80 ± 2.06	6.34 ± 12.26	<0.001
NEXN (mean ± SD)	4.58 ± 11.37	10.52 ± 18.71	0.023
SUN1 (mean ± SD)	1.56 ± 4.28	6.67 ± 18.34	0.006

**Table 2:** Internal benchmarking (top five by OOF AUC). For internal scikit-learn benchmarking across fifteen classical classifiers, the random-forest model was the most accurate to discriminate open-angle glaucoma from cataract. OOF = Out-Of-Fold; AUC = Area Under the Curve; MCC = Matthew’s Correlation Coefficient.

Model	AUC	Precision	Recall	F1 Score	Kappa	MCC
Random Forest	0.839 ± 0.026	0.823	0.916	0.865	0.249	0.268
CatBoost	0.826 ± 0.051	0.833	0.907	0.867	0.301	0.318
Extra Trees	0.824 ± 0.063	0.831	0.933	0.877	0.312	0.351
XGBoost	0.803 ± 0.054	0.828	0.882	0.853	0.269	0.278
Gradient Boosting	0.799 ± 0.036	0.812	0.865	0.835	0.193	0.224

## Data Availability

Restrictions apply to the datasets. The datasets presented in this article are not readily available because the data are part of an ongoing study. Requests to access the datasets should be directed to the corresponding author.

## Supplementary Materials

The following supporting information can be downloaded at: https://www.mdpi.com/article/10.3390/biomedicines13123031/s1, Figure S1: SHAP summary (bar). Mean absolute SHAP values ranking global feature importance; Table S1: Full internal benchmarking (all models, OOF); Table S2: Ablation analysis of autoantibody features under stratified 5-fold out-of-fold (OOF) evaluation with the tuned random-forest; Table S3: Calibration of predicted probabilities: stratified 5-fold OOF ROC–AUC and Brier score for the base model and calibrated models.
