# Supplementary material for: An Exploratory Study of Machine Learning-Based Open-Angle Glaucoma Detection Using Specific Autoantibodies
Source: Biomedicines. 2025 Dec 10;13(12):3031. doi: 10.3390/biomedicines13123031 (PMC12730685; doi:10.3390/biomedicines13123031)
Supplement: Supplementary file 1 [file biomedicines-13-03031-s001.zip › biomedicines-3929525-supplementary.pdf]

Supplement Table S1. Full internal benchmarking (all models, OOF). Full internal benchmarking of all sixteen classifiers under a unified scikit-learn pipeline (OOF evaluation), the Random Forest Classifier was the most accurate to discriminate open angle glaucoma from cataract. SVM=Support Vector Machine, AUC=Area Under the Curve, MCC= Matthews Correlation Coefficient.

Model	OOF AUC	Accuracy	Precision	Recall	F1 Score	Kappa	MCC
Random Forest	0.839 ± 0.026	0.779	0.823	0.916	0.865	0.249	0.268
CatBoost	0.826 ± 0.051	0.785	0.833	0.907	0.867	0.301	0.318
Extra Trees	0.824 ± 0.063	0.799	0.831	0.933	0.877	0.312	0.351
XGBoost	0.803 ± 0.054	0.766	0.828	0.882	0.853	0.269	0.278
Gradient Boosting	0.799 ± 0.036	0.740	0.812	0.865	0.835	0.193	0.224
QDA	0.785 ± 0.066	0.623	0.907	0.571	0.696	0.262	0.315
Light GBM	0.783 ±0.064	0.740	0.813	0.865	0.837	0.191	0.200
Linear SVM	0.771 ± 0.065	0.649	0.902	0.614	0.729	0.280	0.325
AdaBoost	0.762 ± 0.096	0.772	0.846	0.874	0.857	0.280	0.282
Gaussian NB	0.756 ± 0.074	0.571	0.914	0.495	0.638	0.209	0.279
Logistic Regression	0.749 ± 0.085	0.779	0.785	0.983	0.873	0.093	0.125
RBF SVM	0.724 ± 0.181	0.766	0.771	0.991	0.867	−0.012	−0.020
k-NN	0.701 ± 0.147	0.695	0.778	0.849	0.812	0.012	0.006
Ridge Classifier	0.699 ± 0.136	0.565	0.896	0.505	0.641	0.176	0.238
LDA	0.698 ± 0.135	0.773	0.773	1.000	0.872	0.000	0.000
Decision Tree	0.617 ± 0.094	0.766	0.822	0.891	0.855	0.257	0.263
Dummy classifier	0.500 ± 0.000	0.773	0.773	1.000	0.872	0.000	0.000

Supplement Table S2. Ablation analysis of autoantibody features under stratified 5-fold out-of-fold (OOF) evaluation with the tuned random-forest. All metrics are computed from stratified 5-fold out-of-fold predictions under identical preprocessing (median imputation and z-score standardization). ΔAUC is the difference in mean OOF ROC–AUC relative to the "all_features" model. ETNK1 = ethanolamine kinase 1; VMAC = vimentin-type intermediate filament-associated coiled-coil protein; NEXN = nexilin; SUN1 = Sad1 and UNC84 domain containing 1.

Setting	n features	OOF AUC (mean ± SD)	Accuracy	Precision	Recall	F1	ΔAUC
All_features	7	0.852 ± 0.040	0.792	0.833	0.924	0.874	0
drop_etnk1	6	0.828 ± 0.078	0.799	0.827	0.941	0.879	-0.02
drop_nexn	6	0.842 ± 0.061	0.792	0.839	0.907	0.871	-0.01
drop_sun1	6	0.839 ± 0.039	0.766	0.809	0.916	0.858	-0.01
drop_vmac	6	0.749 ± 0.063	0.740	0.777	0.933	0.847	-0.10

Supplement Table S3. Calibration of predicted probabilities: stratified 5-fold OOF ROC–AUC and Brier score for the base model and calibrated models. "base" denotes the uncalibrated tuned random-forest. "sigmoid" and "isotonic" refer to Platt (logistic) and isotonic calibration via CalibratedClassifierCV. OOF = out-of-fold; AUC = area under the ROC curve; SD = standard deviation. Lower Brier scores indicate better overall calibration.

Model	OOF AUC (mean ± SD)	Brier score
base	0.852 ± 0.040	0.134
sigmoid	0.839 ± 0.037	0.143
isotonic	0.835 ± 0.046	0.141

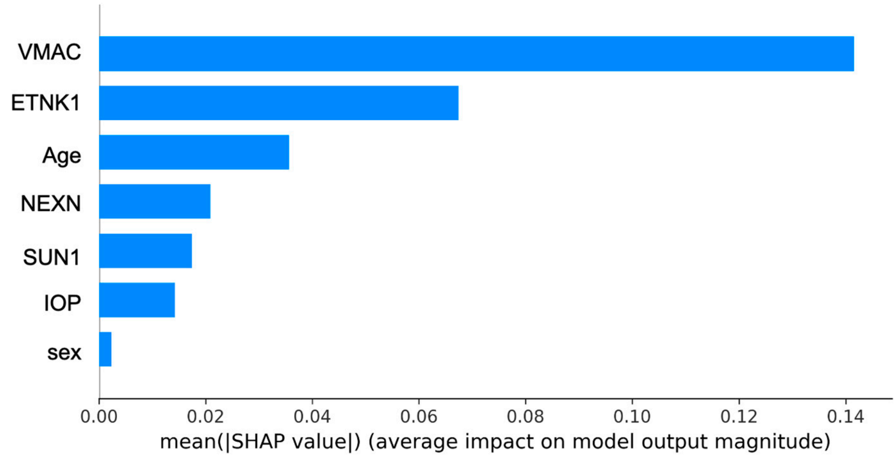

Supplementary Figure S1. SHAP summary (bar). Mean absolute SHAP values ranking global feature importance

VMAC and ETNK1 had the highest average impact on model output, followed by Age and NEXN.

VMAC= vimentin-type intermediate filament-associated coiled-coil protein,

ETNK1=ethanolamine kinase 1, NEXN=nexilin,

SUN1=Sad1 and UNC84 Domain Containing 1, IOP=intra ocular pressure,
